# Efficacy of extracorporeal shock wave combined spinal core decompression for the treatment of patients with femoral head necrosis: A protocol for systematic review and meta-analysis

**DOI:** 10.1097/MD.0000000000020350

**Published:** 2020-05-22

**Authors:** Xiao-feng Qiao, Shi-chen Liu, Yu Xue, Qing-hui Ji

**Affiliations:** a First Ward of Orthopedis Department, First Affiliated Hospital of Jiamusi University; b Department of Obstetrics and Gynecology, Second Affiliated Hospital of Jiamusi University, Jiamusi, China.

**Keywords:** efficacy, extracorporeal shock wave, femoral head necrosis, spinal core decompression

## Abstract

**Background:**

Previous studies have reported that extracorporeal shock wave (EPSW) combined spinal core decompression (SCD) has been used for the treatment of patients with femoral head necrosis (FHN) effectively. However, their results are still inconsistent. Therefore, this study will systematically assess the efficacy and safety of EPSW and SCD for the treatment of patients with FHN.

**Methods:**

This study will systematically search the following databases from inception through March 1, 2020: MEDLINE, Web of Science, Scopus, EMBASE, Cochrane Library, Cumulative Index to Nursing and Allied Health Literature, and China National Knowledge Infrastructure. All searches will be performed without language and publication date restrictions. This study will only include randomized controlled trials investigating the efficacy and safety of EPSW and SCD for the treatment of patients with FHN. Two authors will independently assess all literatures, extract data, and appraise risk of bias. Any confusion between 2 authors will be cleared up by a third author through discussion. RevMan 5.3 software will be utilized to analyze the data and to perform a meta-analysis if necessary.

**Results:**

This study will summarize up-to-date evidence and provide a detailed summary related to the efficacy and safety of EPSW and SCD for the treatment of patients with FHN.

**Conclusion:**

This study may provide helpful evidence to determine whether or not EPSW combined SCD is effective and safety for the treatment of patients with FHN.

**Systematic review registration:**

INPLASY202040092.

## 1 Introduction

Femoral head necrosis (FHN) is a chronic painful bone disease in people aged 20 to 40 years old.^[1–4]^ It is estimated that about 20,000 to 30,000 patients are diagnosed with FHN annually in the USA.^[5]^ It can not be treated effectively and timely, it is more likely to cause femoral neck fractures.^[6–14]^ Published studies have reported that extracorporeal shock wave (EPSW) combined spinal core decompression (SCD) can help to manage patients with FHN.^[15–21]^ However, there is not systematic review that evaluates the efficacy and safety of EPSW and SCD for the treatment of patients with FHN. The present study aims to assess the efficacy and safety of EPSW combined SCD for the treatment of FHN.

## 2 Methods and analysis

### 2.1 Study registration

This protocol has been registered on INPLASY202040092, and it has been organized following the guideline of Preferred Reporting Items for Systematic Reviews and Meta-Analysis Protocol statement.^[22–23]^

### 2.2 Eligibility criteria for study selection

#### 2.2.1 Types of study

We will include randomized controlled trials (RCTs) that evaluated the efficacy and safety of EPSW and SCD for the treatment of patients with FHN. We will exclude literatures that belong to the animal studies, non-clinical trials, uncontrolled clinical trials, and quasi-RCTs.

#### 2.2.2 Types of participant

We will include patients who were diagnosed with FHN, regardless their country, race, gender, and duration and severity of FHN.

#### 2.2.3 Types of intervention

##### 2.2.3.1 Interventions

In the experimental group, all patients must receive EPSW combined SCD therapy alone. Any combined therapies with EPSW or/ and SCD will be excluded.

##### 2.2.3.2 Comparators

In the control group, all participants could undergo any treatments without limitations. However, we will exclude studies that involved treatments of EPSW or/ and SCD.

#### 2.2.4 Types of outcome measurement

Primary outcome is pain intensity (assessed by any pain scales, such as Numerical Rating Scale).

Secondary outcomes are pain, stiffness, and physical function of attacked knee and hip joints (as measured by Western Ontario and McMaster Universities Osteoarthritis Index or other relevant tools); and health-related quality of life (as identified by 36-Item Short Form Health Survey or other related scores), and adverse events.

### 2.3 Literature search

We will systematically search MEDLINE, Web of Science, Scopus, EMBASE, Cochrane Library, Cumulative Index to Nursing and Allied Health Literature, and China National Knowledge Infrastructure. We will search each electronic database from inception through March 1, 2020 without language and publication date limitations. This study will only consider RCTs that explored the efficacy and safety of EPSW and SCD for the treatment of patients with FHN. The search strategy for MEDLINE is created in Table 1. We will also build similar search strategies for other electronic databases.

**Table 1:**
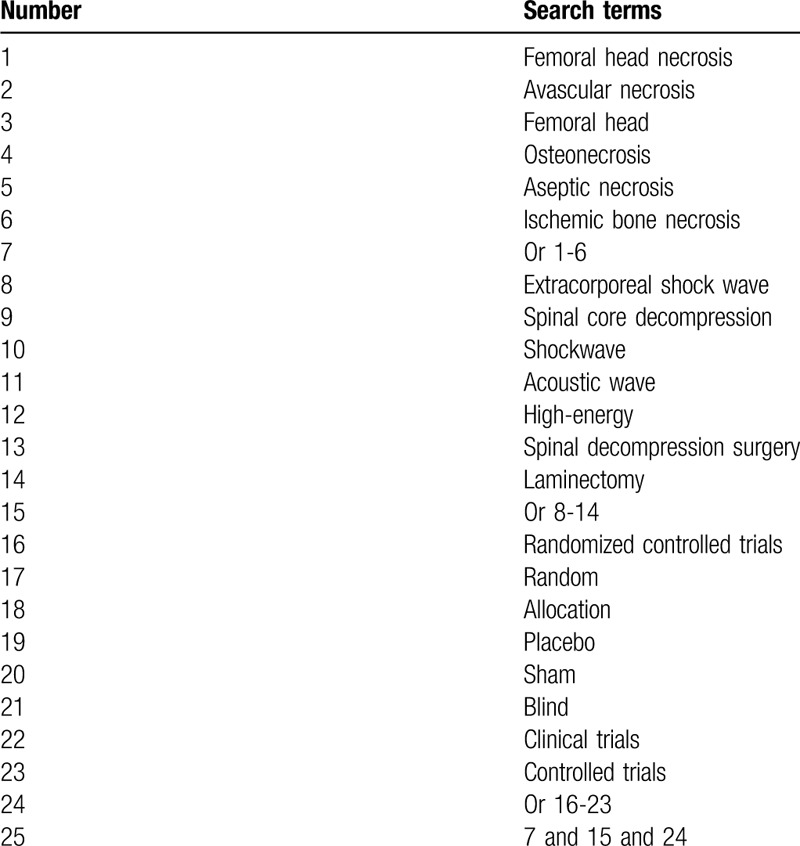
Search strategy utilized for MEDLINE.

In addition, we will investigate other literature sources to avoid missing potential studies, such as conference abstracts and reference lists of related reviews.

### 2.4 Study selection

The titles/abstracts of searched literatures will be scanned by 2 independent authors to identify studies that potentially fulfill the predetermined eligibility criteria, and duplicates and irrelevant records will be removed. The full-text of those potential eligible studies will be obtained and further determined against all inclusion criteria. We will note any excluded studies with reasons and will be listed them in a table. Any inconsistencies between 2 authors will be solved by a third author through discussion. The process of study selection with details is showed in a flowchart.

### 2.5 Data extraction and management

Two independent authors will extract data utilizing predefined data acquisition sheet. Any discrepancies between 2 authors will be resolved by a third author through consultation, and a consensus will be reached. The sheet includes study characteristics (study identification, time of publication, country, et al), study population (country, age, inclusion and exclusion criteria, et al), study design (sample size, details of randomization, blind, et al), intervention and comparison (treatment types, dosage, frequency, et al), outcomes, safety, results, findings, and other related information.

### 2.6 Missing data dealing with

Any insufficient or missing information will be requested from original authors by email or telephone. An intention-to-treat analysis will be applied to analyze outcome data. We will discuss its possible affects on the study findings as a limitation.

### 2.7 Risk of bias assessment

Risk of bias for all included RCTs will be appraised by 2 independent authors using Cochrane Risk of Bias Tool. Each study will be evaluated through 7 aspects and each criteria will be valued as low, unclear or high risk of bias. Differences between 2 authors will be settled through consensus with the help of a third author.

### 2.8 Statistical analysis

#### 2.8.1 Data synthesis

We will use RevMan 5.3 software to analyze the data, and to perform a meta-analysis if necessary. Any dichotomous data (such as incidence of adverse events) will be calculated as risk ratio and 95% confidence intervals, and any continuous data (such as pain intensity) will be rated as mean difference or standardized mean difference and 95% confidence intervals. Statistical heterogeneity will be checked using *I*^*2*^ test. *I*^*2*^ ≤ 50% suggests little or no statistical heterogeneity, and we will employ a fixed-effects model. If sufficient trials are included with little or no statistical heterogeneity, we will consider conducting a meta-analysis. *I*^*2*^ > 50% means obvious heterogeneity, and we will place a random-effects model. A subgroup analysis will be performed to investigate possible sources of remarkable heterogeneity. If necessary, we will also carry out a narrative summary.

#### 2.8.2 Subgroup analysis

If sufficient data is available, a subgroup analysis will be conducted to identify the sources of obvious heterogeneity according to the differences in study and patient characteristics, types of interventions and comparators, and outcomes.

#### 2.8.3 Sensitivity analysis

A sensitivity analysis will be carried out to examine the robustness of the study findings according to the methodological weaknesses and missing data.

#### 2.8.4 Publication bias

A funnel plot and Egger test will be investigated to identify the publication biases if more than 10 RCTs are included.

#### 2.8.5 Summary of evidence

Two authors will independently assess the quality of evidence for each outcome by Grading of Recommendations Assessment, Development, and Evaluation System approach.^[24,25]^ Any opposition will be solved by a consensus through discussion with the help of a third author.

### 2.9 Dissemination and ethics

This study dose not needs ethical approval, since no individual patient data will be obtained. This study will be planed to be published through a peer-reviewed journal.

## 3 Discussion

A growing number of clinical studies have investigated the efficacy and safety of EPSW combined SCD for the treatment of FHN. However, no evidence is presented at evidence-based medicine level. The present work provides a protocol of systematic review of previous clinical trials that examined the efficacy and safety of EPSW combined SCD for the treatment of FHN. The results of this study will timely supply a detailed and summary of the existing evidence of EPSW combined SCD for the treatment of FHN. It will also provide reference and recommendation for clinical practice and future studies.

## Author contributions

**Conceptualization:** Yu Xue, Qing-hui Ji.

**Data curation:** Xiao-feng Qiao, Shi-chen Liu, Qing-hui Ji.

**Investigation:** Qing-hui Ji.

**Formal analysis:** Xiao-feng Qiao, Shi-chen Liu, Yu Xue.

**Methodology:** Xiao-feng Qiao, Shi-chen Liu, Yu Xue.

**Project administration:** Qing-hui Ji.

**Resources:** Xiao-feng Qiao, Shi-chen Liu, Yu Xue.

**Software:** Xiao-feng Qiao, Shi-chen Liu, Yu Xue.

**Supervision:** Qing-hui Ji.

**Validation:** Xiao-feng Qiao, Shi-chen Liu, Qing-hui Ji.

**Visualization:** Xiao-feng Qiao, Yu Xue, Qing-hui Ji.

**Writing – original draft:** Xiao-feng Qiao, Shi-chen Liu, Qing-hui Ji.

**Writing – review and editing:** Xiao-feng Qiao, Shi-chen Liu, Yu Xue, Qing-hui Ji.
